# Tagraxofusp maintenance post-hematopoietic stem cell transplantation provides long-term survival and manageable safety for a patient with blastic plasmacytoid dendritic cell neoplasm

**DOI:** 10.1016/j.lrr.2025.100518

**Published:** 2025-06-12

**Authors:** Qaiser Bashir, Marina Konopleva, Glorette Abueg, Jeremy Ramdial, Chitra Hosing, Samer A. Srour, Amin Alousi, Uday R. Popat, Yago Nieto, Gheath Alatrash, Richard E. Champlin, Elizabeth J. Shpall, Muzaffar Qazilbash, Naveen Pemmaraju

**Affiliations:** a Department of Stem Cell Transplantation and Cellular Therapy, The University of Texas MD Anderson Cancer Center, 1515 Holcombe Blvd, Houston, TX 77030, USA; b Albert Einstein College of Medicine, 1300 Morris Park Ave, Bronx, NY 10461, USA; c Department of Leukemia, The University of Texas MD Anderson Cancer Center, 1515 Holcombe Blvd, Houston, TX 77030, USA

**Keywords:** Allogeneic stem cell transplant, Blastic plasmacytoid dendritic cell neoplasm, BPDCN, Tagraxofusp, CD123

## Abstract

Presented here is the case of a 68-year-old woman with blastic plasmacytoid dendritic cell neoplasm (BPDCN) treated with tagraxofusp (TAG) maintenance therapy post-allogeneic hematopoietic stem cell transplantation (allo-HCT). Prior to allo-HCT, the patient was treated with hydroxyurea and mini-CVD (cyclophosphamide, vincristine, and dexamethasone alternating with methotrexate (Methotrexate) and cytarabine) + venetoclax + TAG for 5 cycles, which induced morphologic complete remission with minimal residual disease. After allo-HCT, the patient had persistent cytogenic abnormalities 45,XX,der(7)add(7)(p13)del(7)(q11.2q22)add(7)(q32),add(12)(p13),-15,del(16)(q23),-17,+22,+2mar[1]/46,XX[19], and was then treated with TAG maintenance therapy at 9 mg/kg on a 28-day cycle for 16 cycles. At mid-treatment (cycle 6 of 16 cycles of TAG) and prior to TAG discontinuation (due to travel burden), the patient was negative for minimal residual disease and was in complete remission. There was no high-grade toxicity or capillary leak syndrome. The patient remains in complete remission as of present day (>17 months CR). This is the first case study illustrating the feasibility of long-term TAG maintenance post-allo-HCT to control BPDCN.

## 1 Introduction

Blastic plasmacytoid dendritic cell neoplasm (BPDCN) is a rare, aggressive hematologic malignancy arising from precursors of plasmacytoid dendritic cells [1]. The true incidence of BPDCN has been difficult to ascertain because of the condition’s rarity and frequently shifting nomenclature (due to improved cytologic characterization) [2]. In the United States, BPDCN incidence is estimated at 0.05 cases per 100,000, with the disease estimated to account for 0.44 % of all hematologic malignancies [3]. BPDCN is most common in adult males (male:female ratio of 3:1) and in older age (mean 67 years old) individuals, although it has been reported in all age groups [2]. The disease typically affects the skin, followed by rapid progression to the bone marrow and peripheral blood, lymph nodes, and eventually multiple organ systems [4,5]. Central nervous system involvement has also been reported [5]. Historically, prognosis and overall survival (OS) are poor, with conventional chemotherapy producing a median OS of only 8–12 months [6]. For those bridged to hematopoietic stem cell transplant (HCT; the treatment goal for fit patients) [7], data from the Center for International Blood and Marrow Transplant Registry (CIBMTR) of 164 patients who received allogeneic HCT (allo-HCT) between 2007–2018 suggest a 5-year OS and disease-free survival rate of 51 % and 44 %, respectively [8].

Recent progress in BPDCN immunohistology has led to the development of targeted therapies, including tagraxofusp (TAG) [4,5]. Nearly all BPDCN cases show notable overexpression of the interleukin-3 receptor (IL-3R) alpha subunit known as CD123 [3]. TAG is a first-in-class CD123-directed therapy comprising a recombinant fusion protein consisting of human interleukin-3 (IL-3) conjugated to a truncated diphtheria toxin payload [9]. TAG delivers its cytotoxic payload to IL-3R-overexpressing cells, including plasmacytoid dendritic cells, leading to the inhibition of BPDCN cell growth [10]. TAG is the only drug approved for the treatment of BPDCN by the US Food and Drug Administration for adults or pediatric patients aged ≥2 years with treatment-naïve or relapsed/refractory BPDCN and by the European Medicines Agency for adults with treatment-naïve BPDCN [10]. These approvals were based on demonstrated efficacy and safety in adults with treatment-naïve and relapsed/refractory BPDCN. Specifically, long-term results of the pivotal phase 1/2 trial and its continued access phase (*N* = 84 efficacy-evaluable patients who received TAG 12 µg/kg/day) showed high rates of durable complete response (CR) and successful bridging to HCT [3,10,11].

As post-transplant relapse among patients with BPDCN bridged from chemotherapy remains common, TAG may be a rational maintenance option in the post-HCT setting [6,8,12,13]. Data from the CIBMTR showed 1- and 5-year cumulative relapse rates of 17 % and 32 % following allo-HCT [8]. Based on these findings and other available data, long-term relapse following chemotherapy and allo-HCT transplant can be estimated at approximately 30 % [6,8,12,13]. To date, no clinical research has assessed the impact of TAG maintenance therapy following HCT on remission/relapse. In 2020, a phase 2 trial (NCT04317781) was initiated to evaluate the safety of TAG in patients with BPDCN after HCT, but was terminated in early 2023 due to slow enrollment, an issue intrinsic to studies of rare diseases [14]. As such, case reports are particularly relevant to rare diseases, wherein therapeutic progress may be hindered by the delay required to enroll large numbers of patients in clinical trials [15]. This case study reports on a patient successfully treated in the phase 2 trial with TAG therapy after allo-HCT. The trial was approved by the institutional review board at the University of Texas MD Anderson Cancer Center, and all patients provided written informed consent.

## 2 Case presentation

This case involved an adult female patient, 68 years old (66 years at time of BPDCN diagnosis), who initially presented in mid-late 2020 with fatigue, cervical lymph node swelling, and thrombocytopenia (platelet count 24,000 µL). The patient had also noticed a purplish, bruise-like lesion on the left forearm. Subsequently, bone marrow biopsy revealed 87 % blasts, and blood examination revealed the following abnormalities: white blood cell (WBC) count, 21.6 × 10^9^/L; blasts in peripheral blood, 78 %; hemoglobin, 6.3 g/dL; platelet count, 13 × 10^9^/L; serum lactate dehydrogenase, 1773 IU/L; and total bilirubin, 1.8 mg/dL (indirect, 1.4 mg/dL; direct, 0.4 mg/dL). Positron emission tomography (PET)-computed tomography (CT) showed no evidence of fluorodeoxyglucose-18 (FDG)-avid disease, while skin biopsy showed superficial perivascular dermatitis and microscopic involvement with BPDCN. Based on lumbar puncture, there was no cerebrospinal fluid involvement.

The patient was treated briefly with hydroxyurea for high WBC count. This was followed by treatment with mini-CVD (dose-attenuated hyperfractionated cyclophosphamide, vincristine, and dexamethasone alternating with methotrexate and cytarabine) + venetoclax, followed by TAG for five 28-day cycles, starting in November 2020. After five treatment cycles, the patient received a matched related donor allo-HCT with FM100 conditioning, as well as graft-versus-host-disease (GVHD) prophylaxis with mini-methotrexate and tacrolimus. At the time of allo-HCT (June 2021), the patient was in morphologic CR, with no evidence of minimal residual disease (MRD) by flow cytometry on bone marrow biopsy, but evidence of low positivity for MRD by cytogenetic analysis. Following allo-HCT, cytogenetic analysis revealed persistent chromosomal abnormalities indicative of residual/persistent disease (Table 1). Flow cytometry was negative for MRD, and there was no evidence of disease per chest, abdomen, and pelvis CT.

**Table 1:** Result of cytogenetic analysis following Allo-HCT, prior to starting tag maintenance therapy.

**24-hour unstimulated culture**	X	**Cells analyzed**	20
**48-hour unstimulated culture**		**Cells karyotyped**	4
**72-hour LPS stimulated culture**	X	**Abnormal cells**	1
**72-hour PHA stimulated culture**		**Normal cells**	19
**72-hour PPC stimulated culture**		**Cells counted**	
**Findings:**	45,XX,der(7)add(7)(p13)del(7)(q11.2q22)add(7)(q32),add(12)(p13),−15,del(16)(q23),−17,+22,+2mar[1]/46,XX[19]		
**Summary:**	Abnormal result showing previously reported abnormalities		
**Interpretation:**	The previously reported chromosomal abnormalities are still recovered in a single cell in the bone marrow indicating residual/persistent disease.		

Starting in November 2021, the patient was treated in the post-transplant setting with TAG 9 µg/kg x 3 days on a 28-day cycle. TAG was administered by intravenous infusion via syringe pump over 15 min. Per-protocol, the patient was to be treated for 24 cycles, with the option to continue based on investigator opinion. However, the patient received TAG for a total of 16 cycles (1.5 years). After Cycle 16 (March 2023), the patient withdrew treatment consent due to her out-of-state travel burden.

Between Cycles 6 and 7 (June 2022), the patient’s disease was in CR; flow cytometry was negative and bone marrow biopsy revealed no morphologic evidence of BPDCN, with cytogenetic analysis negative for mutations (46XX [20]). These tests were repeated prior to discontinuing study treatment in March 2023. At that time, the results remained negative for MRD, and disease was still in CR. As of last follow-up (August 2024), the patient was in remission.

With respect to safety, the patient developed grade 2 acute skin graft-versus-host disease (GVHD) on day 26 post-transplant, which was successfully managed with topical steroids. Immunosuppression was tapered off 6 months after the allo-HCT; however, at 21 months post-transplant, the patient developed moderate chronic skin GVHD, at which point immunosuppressive therapy was resumed. As of last follow-up, the patient’s symptoms related to chronic skin GVHD had substantially improved, and a plan was in place to taper off GVHD therapy. With respect to TAG, the patient did not experience any high-grade toxicity or capillary leak syndrome (CLS). Weight gain was observed at Cycle 5 (Grade 0) and was not clearly related to the study drug. Cycle 6 was delayed due to elevated liver function tests.

## 3 Discussion

In this report of a 68-year-old woman with BPDCN, the patient experienced a clinically meaningful benefit with TAG maintenance therapy post allo-HCT, with disease in complete remission 6 cycles into a total of 16 cycles of TAG treatment and remaining so to date (24 months). This is the first report of TAG used as post allo-HCT maintenance therapy and provides valuable clinical insight into the real-world management of BPDCN. However, as this is only a single case, the findings are not generalizable to all patients treated with allo-HCT for BPDCN, and causal conclusions cannot be drawn. Furthermore, given the low incidence of BPDCN and the relative dearth of effective treatment options prior to the approval of TAG in 2018, optimal treatment sequencing is still being established. Collective, multicenter, and/or collaborative group studies are needed to tackle the issue of slow accrual in rare diseases like BPDCN [2,16,17].

Although there are no other reports of post-HCT maintenance treatment with TAG with which to compare the current results, an analysis of the TAG pivotal trial has shown the safety and feasibility of long-term TAG administration (up to 76 cycles over a median follow-up of 34 months) in patients who achieved CR but did not bridge to HCT [10,11]. In that analysis, most treatment-emergent adverse events (TEAEs) occurred in the first cycle of TAG, with fewer than 1 % of patients experiencing any TEAE after Cycle 4. There was no evidence of cumulative hematologic toxicity, with all but one CLS event occurring in Cycle 1. CLS events were manageable and resolved quickly (median duration of 6 days). In total, 2/18 (11 %) patients who achieved CR and were not bridged to transplant had a prolonged duration of response of 27 and 52 months, respectively, with ongoing therapy [11]. TAG is also being investigated as post-HCT monotherapy maintenance treatment in patients with CD123+ acute myeloid leukemia (AML), myelofibrosis, or chronic myelomonocytic leukemia (NCT05233618) and in combination with azacitidine as post-HCT maintence treatment in patients with CD123+ AML or myelodysplastic syndrome (NCT06498973) [[18], [19], [20]].

## 4 Conclusion

This patient case analysis demonstrates a high-risk patient with BPDCN experiencing clinically meaningful benefit with TAG maintenance therapy after allo-HCT. This patient exhibited both prolonged survival and manageable safety, highlighting the feasibility of long-term TAG maintenance to control BPDCN following allo-HCT. Given the rarity of BPDCN, this research contributes important prospective clinical data.

## Informed consent

The trial (NCT04317781) was approved by the institutional review board at the University of Texas MD Anderson Cancer Center, and all patients provided written informed consent.

## CRediT authorship contribution statement

**Qaiser Bashir:** Conceptualization, Formal analysis, Methodology, Writing – original draft, Writing – review & editing. **Marina Konopleva:** Conceptualization, Formal analysis, Investigation, Writing – original draft, Writing – review & editing. **Glorette Abueg:** Investigation, Writing – review & editing. **Jeremy Ramdial:** Investigation, Writing – review & editing. **Chitra Hosing:** Investigation, Writing – review & editing. **Samer A. Srour:** Investigation, Writing – review & editing. **Amin Alousi:** Investigation, Writing – review & editing. **Uday R. Popat:** Investigation, Writing – review & editing. **Yago Nieto:** Investigation, Writing – review & editing. **Gheath Alatrash:** Investigation, Writing – review & editing. **Richard E. Champlin:** Investigation, Writing – original draft. **Elizabeth J. Shpall:** Investigation, Writing – review & editing. **Muzaffar Qazilbash:** Investigation, Writing – review & editing. **Naveen Pemmaraju:** Conceptualization, Formal analysis, Investigation, Writing – original draft, Writing – review & editing.

## Declaration of competing interest

**Declaration of Funding:** The study from which this case was extracted was funded by Menarini Group.

**Declaration of Financial/Other Relationships: QB:** honoraria from Binaytara Foundation; research funding from Acrotech, GSK, Pfizer, and Stemline. **MK:** Leadership with Immune Oncology (Board of Directors); institutional research funding from Abbvie, Allogene, AstraZeneca, Cellectis, Genentech, Gilead, ImmunoGen, Janssen, MEI Pharma, Pfizer, Precision Biosciences, Rafael Pharmaceuticals, Sanofi Aventis, Stemline Therapeutics; consulting/advisory role with AbbVie, AstraZeneca, Boehringer, F. Hoffman-La Roche, Genentech, Gilead, Janssen, Legend Biotech, MEI Pharma, Redona, Sanofi Aventis, Sellas, Stemline Therapeutics, Vincerx; intellectual property with Reata Pharmaceuticals; participation on a DSMB or advisory board for AbbVie, Auxenion GmbH, Bakx Therapeutics, Dark Blue Therapeutics, F. Hoffman-La Roche, Genentech, Gilead, Stemline Therapeutics, Vincerx. **GA, JR, SAS, AA, URP, YN, REC, and GA** have nothing to disclose. **CH:** grants or contracts from BioLine Rx and Incyte. **EJS:** royalties or licenses from Affimed, Takeda, and RegeNexus; consulting fees from New York Blood Center and Celaid Therapeutics; honoraria from Montefiore Medical Center, AcCELLerate Forum, ASTCT Corporate Council Meeting, Stanford BMT-CT Symposium, and CART Summit; support for attending meetings/travel from Dava Oncology, NMDP, Stanford BMT-CT Symposium, and IDRC; leadership role with NMDP; scientific advisor for Adaptimmune Unlimited, Navan Technologies, Axio Research, Zelluna Immunotherapy, and FibroBiologics. **MQ:** payments from pharmaceutical companies including Amgen, Angiocrine Bioscience, BioLineRx, Janssen, and NexImmune. **NP:** Institutional research grant from US Dept of Defense, NIH/NCI; Consultant/scientific advisory board/speaking for AbbVie, Aplastic Anemia & MDS International Foundation, Aptitude Health, Astellas Pharma US, Blueprint Medicines, Bristol-Myers Squibb Pharmaceuticals, CancerNet, CareDx, Celgene, Cimeio Therapeutics AG, ClearView Healthcare Partners, CTI BioPharma, Curio Science, Dava Oncology, EUSA Pharma, Harborside Press, Imedex, Immunogen, Intellisphere, Karyopharm, Magdalen Medical Publishing, Medscape, Menarini Group, Morphosys, Neopharm, Novartis Pharmaceuticals, OncLive, Pacylex, Patient Power, PeerView Institute for Medical Education, Pharma Essentia, Physician Education Resource (PER); Board of directors/management for Dan’s House of Hope; Leadership with ASH Committee on Communications, ASCO Cancer.Net Editorial Board; Licenses with Karger Publishers; Editorial board (unpaid) for HemOnc Times/Oncology Times.
